# Social support, mental health needs, and HIV risk behaviors: a gender-specific, correlation study

**DOI:** 10.1186/s12889-019-6985-9

**Published:** 2019-05-28

**Authors:** Lin Fang, Deng-Min Chuang, Maria Al-Raes

**Affiliations:** University of Toronto, Factor-Inwentash Faculty of Social Work, 246 Bloor St West, Toronto, Ontario M5S1V4 Canada

**Keywords:** Social support, Mental health, HIV/AIDS, Gender-specific

## Abstract

**Background:**

The HIV/AIDS epidemic continues to threaten the health and wellbeing of millions in the United States and worldwide. Syndemic theory suggests that HIV/AIDS can cooccur with other afflictions. As close to 20% of US adults live with a mental health condition, it is critical to understand the correlation between HIV risk behaviors and mental health needs, as well as protective factors such as social support in intervening the association between mental distress and HIV risk behaviors. Furthermore, as past research has shown mixed results concerning the function of social support on HIV risks by gender, it is important to conduct a gender-specific analysis.

**Methods:**

To assess the relationship between mental health needs, social support, and HIV risk behaviors, and to assess if social support can be a buffer, weakening the effect of mental health needs on HIV risk, in 2018, we analyzed representative, cross-sectional data from 2016 BRFSS collected from 33,705 individuals from four states in the United States, stratified by gender. Weighted logistic regression analyses, adjusted for age, race, marital status, education, and annual income, assessed the correlation between mental health needs, social support, and HIV risk behaviors. Furthermore, interaction analyses were performed to see if social support modifies the slope of mental health needs as a function of HIV risk behaviors.

**Results:**

For both genders, the odds of participating in HIV risk behaviors increase with mental health needs and decrease with the level of social support. Furthermore, social support mitigates the association between mental health needs and HIV risk behavior involvement for males, as males receiving high level of social support have least odds of HIV risk behaviors relative to males receiving low level of social support. Notably, for females, social support does not serve as a buffer against HIV risk behaviors when their mental health needs increase.

**Conclusion:**

The study contributes to the knowledge base of HIV prevention and highlights the important role of mental health and social support against HIV risk behaviors when developing gender-specific prevention strategies.

## Background

The HIV/AIDS epidemic remains a challenge to healthcare systems both in the United States and worldwide and has a profound impact on economics, community, family structure and personal wellbeing across different populations and countries [1–8]. In 2015, over 1.1 million Americans were living with HIV, 15% of whom did not know their HIV diagnosis [9].

Given the gravity of the impact of the HIV/AIDS epidemic, scholars have proposed models to examine risk behaviors that lead to HIV infection as well as factors that can prevent and reduce HIV transmission. Syndemic theory posits that HIV/AIDS often coexists and interacts synergistically with one or more afflictions, which contributes to excessive disease burden [10, 11]. According to Mental Health America [12], over 18%, or 44 million of American adults have a mental health condition. There has been a rising interest in understanding the link between mental health problems and HIV risk behaviors. Specifically, mental health problems and HIV risk behaviors are correlated among men who have sex with men [13–20], drug injection users [21, 22], female sex workers [23], immigrants [24, 25], transgender women [26], and in general population [27–29]. Together, these studies indicate that people with mental health needs may have increased vulnerability to HIV risk behaviors.

Mental health needs have been shown to differ by gender. Large-scale studies [e.g., 30, 31] have found that females are prone to experience internalizing mental disorders, while externalizing disorders and substance use disorders are more common among males. However, gender differences are less pronounced in younger cohorts for conditions such as depression and substance use [31] or when mental disorders are combined [30]. Studies have also considered gender differences in the association of mental health needs and HIV infection. Anxiety and depression were more likely to co-occur with HIV among heterosexual males living with HIV, while substance use was more likely to co-occur with HIV among heterosexual females living with HIV [32]. Several studies have indicated that females who had any mood or anxiety disorder [33] or alcohol use [34] were more likely to be involved in high risk sexual behaviors than females with no mental health concerns and alcohol use. Nonetheless, both females and males who had greater mental health needs were more likely to have had HIV risk behaviors, such as having more transactional sex, having more than one sex partner [35–37], and exhibiting needle sharing behaviors in the past 30 days [35].

Studies have examined the role of social support, an important interpersonal factor, in protecting people against HIV risks. A systematic review of 41 studies on social support and HIV risk behaviors concluded that a higher level of social support was associated with fewer HIV risk behaviors among general adults as well as female sex workers and people living with HIV/AIDS [38]. Furthermore, the buffering hypothesis [39] suggests that emotional support gained from the social networks can serve as a buffer, protecting the individuals in the face of stressful events and increasing coping. In a survey study that involved 157 female sex workers in Croatia, the researchers identified that social support attenuated the association between victimization and HIV risks (i.e., STI diagnosis) and that female sex workers had highest odds of sexual risk when they had little or no social support, compared to those had moderate or high support [23].

Past research has also highlighted the importance of considering gender differences when understanding the role of social support, and in the correlation between social support and HIV risk behaviors. Compared to females, males perceive to have less social support [40] and are less satisfied with their social support [41]. As well, there are mixed findings concerning gender-specific relationship between social support and HIV risks. For example, a study of individuals attending venues serving alcohol in South Africa [42] found that social support was correlated with fewer sexual risk behaviors for females, but not for males. Contrarily, in a study of drug injection users in Sichuan, China [43], the researchers did not observe a relationship between social support and HIV risk behaviors among female users, but identified that decreased social support was correlated with increased HIV risk behaviors among male users. Among adolescents, perceived support from friends was associated with lower sexual risk behaviors among female adolescents, while perceived support from family, not friends, was associated with lower sexual risk behaviors among male adolescents [44]. Lastly, in a study of Latino Immigrants in New Orleans [45], gender difference was not found in the association between social support and HIV risk behaviors in that social support served as a protective factor for both genders.

Indeed, while the current evidence has consistently pointed to a positive relationship between mental health needs and HIV risks, the function of social support on HIV risk behaviors is less clear when stratified by gender. Moreover, the majority of past studies examining the role of social support in mental health and HIV risks have been based on specific community populations [38], and research evidence drawn from population-based data is lacking. Given that HIV/AIDS and mental health are both critical public health issues, it is important to use population-based data to further understand whether these conditions co-occur and whether social support can serve as a buffer. Using a population-based data and gender-specific analysis, the current study will test three hypotheses: 1) mental health needs are positively associated with HIV risk behaviors, 2) social support is negatively correlated with HIV risk behaviors, and 3) social support moderates the association between mental health needs and HIV risk behaviors.

## Methods

We analyzed 2016 Behavioral Risk Factor Surveillance System (BRFSS) data from Louisiana, Michigan, Rhode Island, and Tennessee. The BRFSS is a representative, telephone survey of health conditions and behavioral risk factors among adults aged 18 and older who live in the United States. The cross-sectional survey contains core modules as well as optional modules that states can choose to include [46]. The four states this study comprises were the only ones that opted in to use the optional emotional and social support module in 2016. Data from these states reflected a total number of 33,705 individuals.

### Measures

Mental health status: Participants were asked, “Now thinking about your mental health, which includes stress, depression and problems with emotions, for how many days during the past 30 days was your mental health not good.” The response was dichotomized as 0 = “none” and 1 = “1 or more days”.

Social support: Participants were asked one question, “how often do you get the social and emotional support you need” and indicated their response on a 5-point Likert scale from “never” to “always”. We recoded the variable into low (1 = “never” or “rarely”), medium (2 = “sometimes” or “usually”), and high (3 = “always”) levels of social support.

HIV risk behaviors: In a single question, participants were asked to indicate if they have engaged in any of the following activities in the past year: “You have used intravenous drugs”, “You have been treated for a sexually transmitted or venereal disease”, “You have given or received money or drugs in exchange for sex”, and “You had anal sex without a condom.” (0 = “No”, 1 = “Yes”).

Demographic information: Participants provided information on age, gender, marital status, education, and annual income.

### Statistical analysis

Bivariate analyses were used to examine participants’ descriptive information by gender. Step-wise logistic regressions were used to assess the correlates of HIV risk behaviors. We created gender-specific models to assess the contribution of mental health needs and social support to HIV risk behaviors. Data were weighted using the weight variable designed by BRFSS that took into consideration study designs and population’s demographic information including age, race/ethnicity, sex, marital status, education, ownership of home, and ownership of telephone [47].

To evaluate the associations between mental health needs, social support, and HIV behaviors (hypotheses 1 and 2), we entered demographic variables in the first step as controls, and mental health needs and social support in the second step. To assess the moderation role of social support (hypothesis 3), we included the interaction term of mental health needs X social support in the final step [48]. ModGraph [49] was used to depict the moderation patterns when the interaction term was significant. All analyses were performed in IBM SPSS Statistics 24 [50]. Due to multiple testings, we used Bonferroni correction [51] and adjusted the study’s critical value at 0.0125 (0.05/4).

## Results

Table 1 shows the descriptive data of study variables. Weighted estimates suggest that, compared to males, a greater proportion of females are older, not married, received more education, but have lower income (all *p*’s < .0001). Females also indicate to have more mental health needs than males (40.3% vs. 29.5%; *p* < .0001). While a greater percentage of females than males receive medium level of social support, slightly more males consider themselves receiving low level of social support than females (10.8% vs. 7.9%; *p* < .0001). Over 7% of males had engaged in HIV risk behaviors in the 12 months, higher than 4.8% of females (*p*’s < .0001).

**Table 1:** Unweighted and weighted data of participant characteristics, mental health needs, social support and HIV risk, stratified by gender (*N* = 33,705)

	Male			Female		
	Unweighted (*n* = 14,796)		Weighted	Unweighted (*n* = 18,909)		Weighted
	*n*	%	%	*n*	%	%
Age (years)						
18–24	1098	7.4	13.1	878	4.6	11.9
25–34	1747	11.8	17.9	1739	9.2	16.8
35–44	1860	12.6	16.5	2096	11.1	15.8
45–54	2504	16.9	17.1	3142	16.6	16.6
55–64	3346	22.6	17.0	4320	22.8	17.1
65–99	4241	28.7	18.3	6734	35.6	21.8
Race						
Non-Hispanic White	12,224	84.0	74.7	15,549	83.4	74.5
Hispanic or non-White	2322	16.0	25.3	3104	16.6	25.5
Marital status						
Not married	5681	38.7	42.7	8895	47.4	47.8
Married or common law	9012	61.3	57.3	9884	52.6	52.2
Education						
Less than high school	1060	7.2	14.2	1324	7.0	12.6
High school or GED diploma	4137	28.1	32.5	5124	27.2	29.6
Some college	4084	27.7	29.1	5405	28.7	32.2
College or graduate degree	5463	37.1	24.2	6983	37.1	25.6
Annual income (in US$)						
< 15,000	903	7.0	8.2	1665	10.7	12.3
15,000 – 24,999	1724	13.4	15.1	2798	18.1	20.5
25,000 – 49,999	3183	24.7	25.9	3959	25.6	25.2
50,000 – 74,999	2263	17.6	16.9	2437	15.7	15.0
> = 75,000	4809	37.3	33.9	4632	29.9	27.0
Current MH needs						
Has need	3841	26.4	29.5	6598	35.5	40.3
No need	10,731	73.6	70.5	11,995	64.5	59.7
Social support						
Low	1211	9.7	10.8	1143	6.9	7.9
Medium	4119	33.1	33.2	6015	36.6	37.4
High	7130	57.2	56.1	9295	56.5	54.7
HIV risk						
Yes	673	4.9	7.4	427	2.4	4.8
No	12,924	95.1	92.6	17,071	97.6	95.2

Note. Weighted analyses suggests that males and females are differed in all demographic variables (*p* < .0001) except for race

Table 2 presents gender-specific associations between mental health needs, perceived social support, and HIV risk behaviors, adjusted for demographic variables. For both genders, HIV risk behaviors are associated with greater mental health needs and lower level of perceived social support. Compared to their counterparts who did not have mental health needs in the past 30 days, males who did have mental health needs have a 49% odds increase (95% CI = 1.24, 1.78, *p* < .0001) in engaging in HIV risk behaviors, and females have a 93% odds increase (95% CI = 1.55, 2.38, *p* < .0001). Conversely, for both males and females, those who reported to have medium (males: adjusted odds ratio (AOR) = 0.54, 95% CI = 0.41, 0.70, *p* < .0001; females: AOR = 0.55, 95% CI, 0.41, 0.75, *p* < .0001) or high levels of social support (males: AOR = 0.52; 95% CI = 0.41, 0.67, *p* < .0001; females: AOR = 0.45; 95% CI = 0.33, 0.62, *p* < .0001) are almost twice less likely to be involved in HIV risk behaviors.

**Table 2:** Multiple logistic regression models of mental health needs and social support as a function of HIV risk behaviors, stratified by gender

	Male			Female		
	AOR^a^	95% CI	*p*	AOR	95% CI	*p*
Age (years)						
18–24	1.00	Ref		1.00	Ref	
25–34	0.92	0.73–1.16	.491	1.02	0.79–1.32	.891
35–44	0.40	0.31–0.53	<.0001	0.44	0.32–0.59	<.0001
45–54	0.15	0.11–0.22	<.0001	0.18	0.12–0.26	<.0001
55–64	0.16	0.11–0.23	<.0001	0.09	0.05–0.14	<.0001
65–99	0.51	0.03–0.09	<.0001	0.05	0.03–0.09	<.0001
Race						
Non-Hispanic White	1.00	Ref		1.00	Ref	
Hispanic or non-White	1.32	1.09–1.60	.005	1.19	0.97–1.47	.103
Marital status						
Not married	1.00	Ref		1.00	Ref	
Married or common law	0.57	0.47–0.70	<.0001	0.43	0.34–0.55	<.0001
Education						
Less than high school	1.00	Ref		1.00	Ref	
High school or GED diploma	0.72	0.56–0.94	.016	1.51	1.07–2.15	.021
Some college	0.80	0.62–1.05	.112	1.14	0.79–1.64	.478
College or graduate degree	0.62	0.45–0.84	.002	0.99	0.66–1.48	.951
Income (US$)						
< 15,000	1.00	Ref		1.00	Ref	
15,000 – 24,999	1.65	1.15–2.36	.007	0.89	0.66–1.19	.413
25,000 – 49,999	1.37	0.97–1.95	.075	0.80	0.59–1.09	.155
50,000 – 74,999	1.26	0.86–1.86	.239	0.77	0.53–1.11	.163
> = 75,000	1.39	0.97–1.99	.077	0.67	0.46–0.97	.035
Mental health needs						
No	1.00	Ref		1.00	Ref	
Yes	1.49	1.24–1.78	<.0001	1.93	1.55–2.38	<.0001
Emotional support						
Low	1.00	Ref		1.00	Ref	
Medium	0.54	0.41–0.70	<.0001	0.55	0.41–0.75	<.0001
High	0.52	0.41–0.67	<.0001	0.45	0.33–0.62	<.0001

*Note:* Both models were adjusted for participant age, race, income, educational level, marital status. The Bonferroni-corrected *p* value is set at .025

^a^AOR, adjusted odds ratio

**Fig. 1:**
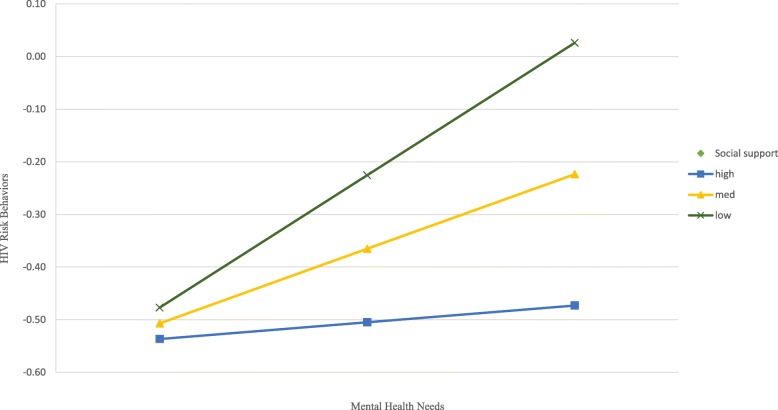
Social support as a moderator between mental health needs and HIV risk behaviors among males

We further tested the moderating role of social support on the association between mental health needs and HIV risk behaviors by introducing the interaction term mental health needs X emotional support in the logistic regression models. While the interaction term was not significant in the model for females, it was significant in male’s model (AOR = 0.70, 95% CI = 0.55, 0.90, *p* = .006). Shown in Fig. 1, the slope of HIV risk behaviors as a function of mental health needs is the steepest among males who have low social support, followed by those who have medium level of social support, and high level of social support, respectively. In other words, males who have low level of social support have the greater odds of engaging in HIV risk behaviors when comparing with those who have medium or high level of social support.

## Discussion

The present study uses retrospective population data to examine the associations between mental health needs, social support and HIV risk behaviors. Our findings suggest that, compared to males, females have higher mental health needs, have higher perception of social support, and practice lower HIV risk behaviors. Furthermore, regardless of gender, greater mental health needs are associated with elevated HIV risk behaviors. While higher social support is connected with fewer HIV risk behaviors for both genders, social support only functions as a moderator between mental health needs and HIV risk behaviors among males, but not among females. Study results contribute to the body of knowledge on the roles of mental health needs and social support related to HIV risk behaviors, underscoring the need to tailor HIV prevention programs with enhancing social support elements for males with mental health needs, as well as the need to continue exploring other mechanisms that can intervene the association between mental health needs and risky sexual behaviors for females.

More females report current mental health needs than males in our study. While this finding corroborates those of previous studies [52, 53], literature also suggests that males are less likely than females to recognize and report their mental health needs and, as a result, rarely access mental health services and treatment [54–56]. It is critical to further understand gender-specific mental health needs as well as help-seeking patterns and decision process, so that targeted strategies that encourage service utilization can be developed.

Furthermore, although females report greater mental health needs than males, individuals with poorer mental health status, regardless of gender, have increased HIV risk behaviors compared to those without. The results further demonstrate the co-occurrence of mental health issues and HIV risk behaviors and are consistent with those of prior studies [21, 44, 57–61]. The most commonly reported mental health needs among people living with HIV are depression or depressive symptoms, anxiety, and suicidal attempts [62, 63]. It is possible that mental health issues can interfere with the individual’s cognitive functioning and affect the decision to practice safer sexual behaviors [60].

The present results show that a higher level of perceived social support is associated with fewer HIV risk behaviors. In addition, perceived social support significantly moderates the relationships between mental health needs and HIV risk behaviors for males, where the relationship between mental health status and HIV risk behaviors is the strongest among males reporting the lowest level of social support, and the weakest among males reporting the highest level of social support. These findings lend support to previous literature in that, when faced with limited social support, males who are psychologically vulnerable may engage in HIV risk behaviors in order to obtain immediate physical intimacy or emotional gratification [64]. Traditional masculine gender roles, such as those emphasizing achievement, autonomy, and emotional control, can not only diminish males’ perception of their mental health needs and inhibit help-seeking behaviors and social support, but also increase risk-taking behaviors [65, 66]. To decrease males’ mental health needs and HIV risk behaviours, prevention programs and services targeting males should consider ways to encourage social and emotional support.

To our surprise, social support does not show to affect the interplay between mental health and HIV risks for females in the same way it did for males. That social support does not show to have a buffering role in HIV vulnerability among females is contradictory to findings from past studies. As these studies (e.g., [23, 67]) focused on female sex workers, rather than females drawn from a general population, the difference in results may be attributed to the variation of study samples. Literature has also suggested that varied sources of social and emotional support (e.g., informal vs. formal) and types of social networks (e.g., family, partners, friends) can result in different effect on HIV risk behaviors for females [38]. Furthermore, there needs to be a distinction concerning the quality of social relationships, as a supportive relationship can generate different outcomes compared to a toxic one.

The study has several limitations. Due to the cross-sectional nature of BRFSS, study results are only correlational. In addition, as indicated earlier, key study variables were measured with a single item, which lacks specificity. While the brevity increases the ease of administration for a large health surveillance survey, it nevertheless constrains the interpretation of study results. Future population-based studies should further unpack these constructs to encourage more comprehensive analyses. Lastly, while BRFSS provided population-based, representative data, only four states that used the emotional and social support module were included in this study. As a result, study findings cannot be generalized to the entire population in the United States.

## Conclusion

To our knowledge, this study is among the few studies that use representative, population-based data to test buffering hypothesis in the context of HIV risks. The study discerns gender-specific mechanisms between mental health needs and HIV risks, providing empirical evidence of the role of mental health needs and social support in HIV risk behaviors. HIV prevention should consider gender differences, look into how to support people with mental health needs, and explore ways to strengthen social support for those who are at risk for HIV infections.

## Abbreviations

AIDS: Acquired Immune Deficiency Syndrome
AOR: Adjusted odds ratio
BRFSS: The Behavioral Risk Factor Surveillance System
HIV: Human Immunodeficiency Virus
IBM: International Business Machines
SPSS: Statistical Package for the Social Sciences
STI: Sexually Transmitted Infection

## References

[1] Alkenbrack Batteh SE, Forsythe S, Martin G, Chettra T (2008). Confirming the impact of HIV/AIDS epidemics on household vulnerability in Asia: the case of Cambodia. AIDS.

[2] Enrico L, Bernhard S (2009). The impact of HIV/AIDS on economic growth in sub-Saharan Africa. South African Journal of Economics.

[3] Gardner LK, Lee SH (2010). The Impact of HIV/AIDS on health capital and economic growth: A panel study of 38 countries from 1999–2005. International Journal of Management.

[4] Guo Y, Li X, Sherr L (2012). The impact of HIV/AIDS on children's educational outcome: a critical review of global literature. AIDS Care.

[5] Ji G, Li L, Lin C, Sun S (2007). The impact of HIV/AIDS on families and children - a study in China. AIDS.

[6] Oramasionwu CU, Daniels KR, Labreche MJ, Frei CR (2011). The environmental and social influences of HIV/AIDS in sub-Saharan Africa: a focus on rural communities. Int J Environ Res Public Health.

[7] Ssengonzi R (2009). The impact of HIV/AIDS on the living arrangements and well-being of elderly caregivers in rural Uganda. AIDS Care.

[8] Wig N, Lekshmi R, Pal H, Ahuja V, Mittal CM, Agarwal SK (2006). The impact of HIV/AIDS on the quality of life: a cross sectional study in North India. Indian J Med Sci.

[9] Basic statistics [https://www.cdc.gov/hiv/statistics/overview/ataglance.html].

[10] Singer M (1996). A dose of drugs, a tough of violence, a case of AIDS: Conceptualiziong the SAVA syndemic. Free Inquiry.

[11] Stall R, Mills TC, Williamson J, Hart T, Greenwood G, Paul J, Pollack L, Binson D, Osmond D, Catania JA (2003). Association of co-occurring psychosocial health problems and increased vulnerability to HIV/AIDS among urban men who have sex with men. Am J Public Health.

[12] The states of mental health in America 2019 [http://www.mentalhealthamerica.net/issues/state-mental-health-america].

[13] Halkitis PN, Moeller RW, Siconolfi DE, Storholm ED, Solomon TM, Bub KL (2013). Measurement model exploring a syndemic in emerging adult gay and bisexual men. AIDS Behav.

[14] Mimiaga MJ, O'Cleirigh C, Biello KB, Robertson AM, Safren SA, Coates TJ, Koblin BA, Chesney MA, Donnell DJ, Stall RD (2015). The effect of psychosocial syndemic production on 4-year HIV incidence and risk behavior in a large cohort of sexually active men who have sex with men. J Acquir Immune Defic Syndr.

[15] Pitpitan EV, Smith LR, Goodman-Meza D, Torres K, Semple SJ, Strathdee SA, Patterson TL (2016). "Outness" as a moderator of the association between syndemic conditions and HIV risk-taking behavior among men who have sex with men in Tijuana, Mexico. AIDS Behav.

[16] Santos GM, Do T, Beck J, Makofane K, Arreola S, Pyun T, Hebert P, Wilson PA, Ayala G (2014). Syndemic conditions associated with increased HIV risk in a global sample of men who have sex with men. Sex Transm Infect.

[17] Storholm ED, Halkitis PN, Siconolfi DE, Moeller RW (2011). Cigarette smoking as part of a syndemic among young men who have sex with men ages 13-29 in new York City. J Urban Health.

[18] Tulloch TG, Rotondi NK, Ing S, Myers T, Calzavara LM, Loutfy MR, Hart TA (2015). Retrospective reports of developmental stressors, syndemics, and their association with sexual risk outcomes among gay men. Arch Sex Behav.

[19] Mustanski B, Garofalo R, Herrick A, Donenberg G (2007). Psychosocial health problems increase risk for HIV among urban young men who have sex with men: preliminary evidence of a syndemic in need of attention. Ann Behav Med.

[20] Reisner SL, Mimiaga MJ, Skeer M, Bright D, Cranston K, Isenberg D, Bland S, Barker TA, Mayer KH (2009). Clinically significant depressive symptoms as a risk factor for HIV infection among black MSM in Massachusetts. AIDS Behav.

[21] German D, Latkin CA (2012). Boredom, depressive symptoms, and HIV risk behaviors among urban injection drug users. AIDS Behav.

[22] Moeller RW, Halkitis PN, Surrence K (2011). The interplay of syndemic production and serosorting in drug-using gay and bisexual men. Journal of Gay & Lesbian Social Services.

[23] Štulhofer A, Sinković M, Božić J, Baćak V (2017). Victimization and HIV risks among Croatian female sex workers:exploring the mediation role of depressiveness and the moderation role of social support. Violence Against Women.

[24] Gonzalez-Guarda RM, Florom-Smith AL, Thomas T (2011). A syndemic model of substance abuse, intimate partner violence, HIV infection, and mental health among Hispanics. Public Health Nurs.

[25] Gonzalez-Guarda RM, McCabe BE, Vermeesch AL, Cianelli R, Florom-Smith AL, Peragallo N (2012). Cultural phenomena and the sydemic factors: substance abuse, violence, HIV, and depression among Hispanic women. Annals of anthropological practice.

[26] Chakrapani V, Newman PA, Shunmugam M, Logie CH, Samuel M (2017). Syndemics of depression, alcohol use, and victimisation, and their association with HIV-related sexual risk among men who have sex with men and transgender women in India. Glob Public Health.

[27] Mthembu JC, Mabaso MLH, Khan G, Simbayi LC (2017). Prevalence of psychological distress and its association with socio-demographic and HIV-risk factors in South Africa: findings of the 2012 HIV prevalence, incidence and behaviour survey. SSM - Population Health.

[28] Chipimo PJ, Fylkesnes K (2009). Mental distress in the general population in Zambia: impact of HIV and social factors. BMC Public Health.

[29] Lundberg P, Rukundo G, Ashaba S, Thorson A, Allebeck P, Östergren P-O, Cantor-Graae E (2011). Poor mental health and sexual risk behaviours in Uganda: a cross-sectional population-based study. BMC Public Health.

[30] Boyd A, Van de Velde S, Vilagut G, de Graaf R, O’Neill S, Florescu S, Alonso J, Kovess-Masfety V (2015). Gender differences in mental disorders and suicidality in Europe: results from a large cross-sectional population-based study. J Affect Disord.

[31] Seedat S, Scott KM, Angermeyer MC, Berglund P, Bromet EJ, Brugha TS, Demyttenaere K, de Girolamo G, Haro JM, Jin R (2009). Cross-national associations between gender and mental disorders in the World Health Organization world mental health surveys. Arch Gen Psychiatry.

[32] Tsuyuki K, Pitpitan EV, Levi-Minzi MA, Urada LA, Kurtz SP, Stockman JK, Surratt HL (2017). Substance use disorders, violence, mental health, and HIV: differentiating a syndemic factor by gender and sexuality. AIDS Behav.

[33] Cook JA, Burke-Miller JK, Steigman PJ, Schwartz RM, Hessol NA, Milam J, Merenstein DJ, Anastos K, Golub ET, Cohen MH. Prevalence, comorbidity, and correlates of psychiatric and substance use disorders and associations with HIV risk behaviors in a multisite cohort of women living with HIV. AIDS Behav. 2018. 10.1007/s10461-018-2051-3 PMC6153984 29460130

[34] Pitpitan EV, Kalichman SC, Eaton LA, Sikkema KJ, Watt MH, Skinner D: Gender-based violence and HIV sexual risk behavior: alcohol use and mental health problems as mediators among women in drinking venues, Cape Town *Social science & medicine (1982)* 2012, 75(8):1417–1425. 10.1016/j.socscimed.2012.06.020 PMC3425436 22832324

[35] Corsi KF, Garver-Apgar C, Booth RE (2014). Gender differences in HIV risk and mental health among methamphetamine users. Drug Alcohol Depend.

[36] Nduna Mzikazi, Jewkes Rachel K, Dunkle Kristin L, Shai Nwabisa P Jama, Colman Ian (2010). Associations between depressive symptoms, sexual behaviour and relationship characteristics: a prospective cohort study of young women and men in the Eastern Cape, South Africa. Journal of the International AIDS Society.

[37] Sherr L, Clucas C, Lampe F, Harding R, Johnson M, Fisher M, Anderson J, Edwards S, Team S (2012). Gender and mental health aspects of living with HIV disease and its longer-term outcomes for UK heterosexual patients. Women Health.

[38] Qiao S, Li X, Stanton B (2014). Social support and HIV-related risk behaviors: a systematic review of the global literature. AIDS Behav.

[39] Cohen S, Wills TA (1985). Stress, social support, and the buffering hypothesis. Psychol Bull.

[40] Osman A, Lamis DA, Freedenthal S, Gutierrez PM, McNaughton-Cassill M (2014). The multidimensional scale of perceived social support: analyses of internal reliability, measurement invariance, and correlates across gender. J Pers Assess.

[41] Semple Shirley J, Patterson Thomas L, Straits-Troster Kristy, Atkinson J. H., McCutchan J. A., Grant Igor, Group HNRC (1996). Social and Psychological Characteristics of HIV-Infected Women and Gay Men. Women & Health.

[42] Velloza J, Watt MH, Abler L, Skinner D, Kalichman SC, Dennis AC, Sikkema KJ (2017). HIV-risk behaviors and social support among men and women attending alcohol-serving venues in South Africa: implications for HIV prevention. AIDS Behav.

[43] Choi SY, Cheung YW, Chen K (2006). Gender and HIV risk behavior among intravenous drug users in Sichuan Province, China. Soc Sci Med.

[44] Ramiro MT, Teva I, Bermúdez MP, Buela-Casal G (2013). Social support, self-esteem and depression: relationship with risk for sexually transmitted infections/HIV transmission. Int J Clin Health Psychol.

[45] Althoff MD, Theall K, Schmidt N, Hembling J, Gebrekristos HT, Thompson MM, Muth SQ, Friedman SR, Kissinger P (2017). Social support networks and HIV/STI risk behaviors among Latino immigrants in a new receiving environment. AIDS Behav.

[46] Centers for Disease Control and Prevention (2017). Behavioral Risk Factor Surveillance System. Overview: BRFSS 2016.

[47] Centers for Disease and Prevention (2017). Behavioral risk factor surveillance system: weighting BRFSS data. BRFSS 2016.

[48] Jose PE. Doing statistical mediation and moderation. New York: Guilford Press; 2013.

[49] Jose PE: ModGraph-I: A programme to compute cell means for the graphical display of moderational analyses: The internet version, Version 3.0. Victoria University of Wellington, Wellington, New Zealand. Retrievedon Oct 25, 2018 from https://psychology.victoria.ac.nz/modgraph/. In*.*; 2013.

[50] Inc IBMSPSS (2016). SPSS 24.0 for Windows.

[51] Bland JM, Altman DG (1995). Multiple significance tests: the Bonferroni method. BMJ.

[52] Chen G, Gueta K (2016). Childhood abuse and mental health problems: does gender matter?. Psychiatr Q.

[53] Needham B, Hill TD (2010). Do gender differences in mental health contribute to gender differences in physical health?. Soc Sci Med.

[54] Gouwy A, Christiaens W, Bracke P (2008). Mental health services use in the general Belgian population: estimating the impact of mental health and social determinants. Archives of Public Health.

[55] Webster JM, Rosen PJ, Krietemeyer J, Mateyoke-Scrivner A, Staton-Tindall M, Leukefeld C (2006). Gender, mental health, and treatment motivation in a drug court setting. J Psychoactive Drugs.

[56] Yan J, Dannerbeck A (2011). Exploring the relationship between gender, mental health needs, and treatment orders in a metropolitan juvenile court. J Child Fam Stud.

[57] Bekele T, Collins EJ, Maunder RG, Gardner S, Rueda S, Globerman J, Le TL, Hunter J, Benoit A, Rourke SB (2018). Childhood adversities and physical and mental health outcomes in adults living with HIV: findings from the Ontario HIV Treatment Network cohort study. AIDS Research and Treatment.

[58] Ssewamala FM, Bermudez LG, Neilands TB, Mellins CA, McKay MM, Garfinkel I, Sensoy Bahar O, Nakigozi G, Mukasa M, Stark L (2018). Suubi4Her: a study protocol to examine the impact and cost associated with a combination intervention to prevent HIV risk behavior and improve mental health functioning among adolescent girls in Uganda. BMC Public Health.

[59] Hill LM, Moody J, Gottfredson NC, Kajula LJ, Pence BW, Go VF, Maman S (2018). Peer norms moderate the association between mental health and sexual risk behaviors among young men living in Dar Es Salaam, Tanzania. Soc Sci Med.

[60] Gerbi GB, Habtemariam T, Robnett V, Nganwa D, Tameru B (2012). Psychosocial factors as predictors of HIV/AIDS risky behaviors among people living with HIV/AIDS. Journal of AIDS and HIV Research.

[61] Safren SA, Reisner SL, Herrick A, Mimiaga MJ, Stall RD (2010). Mental health and HIV risk in men who have sex with men. J Acquir Immune Defic Syndr.

[62] Spies G, Archibald SL, Fennema-Notestine C, Sareen J, Seedat S, Afifi TO (2012). Mental health outcomes in HIV and childhood maltreatment: a systematic review. Syst Rev.

[63] Chuah FLH, Haldane VE, Cervero-Liceras F, Ong SE, Sigfrid LA, Murphy G, Watt N, Balabanova D, Hogarth S, Maimaris W, et al. Interventions and approaches to integrating HIV and mental health services: a systematic review. Health Policy Plan. 2017:1–21. 10.1093/heapol/czw169 PMC5886062 29106512

[64] Saleh LD, van den Berg JJ, Chambers CS, Operario D (2016). Social support, psychological vulnerability, and HIV risk among African American men who have sex with men. Psychol Health.

[65] Barbee AP, Cunningham MR, Winstead BA, Derlega VJ, Gulley MR, Yankeelov PA, Druen PB (1993). Effects of gender role expectations on the social support process. J Soc Issues.

[66] Houle J, Mishara BL, Chagnon F (2008). An empirical test of a mediation model of the impact of the traditional male gender role on suicidal behavior in men. J Affect Disord.

[67] Rössler W, Koch U, Lauber C, Hass A-K, Altwegg M, Ajdacic-Gross V, Landolt K (2010). The mental health of female sex workers. Acta Psychiatr Scand.

